# Prior Inoculation with Type B Strains of *Francisella tularensis* Provides Partial Protection against Virulent Type A Strains in Cottontail Rabbits

**DOI:** 10.1371/journal.pone.0140723

**Published:** 2015-10-16

**Authors:** Vienna R. Brown, Danielle R. Adney, Francisco Olea-Popelka, Richard A. Bowen

**Affiliations:** 1 Department of Microbiology, Immunology, and Pathology, Colorado State University, Fort Collins, Colorado, 80523, United States of America; 2 Department of Clinical Sciences, Colorado State University, Fort Collins, Colorado, 80523, United States of America; Albany Medical College, UNITED STATES

## Abstract

*Francisella tularensis* is a highly virulent bacterium that is capable of causing severe disease (tularemia) in a wide range of species. This organism is characterized into two distinct subspecies: *tularensis* (type A) and *holarctica* (type B) which vary in several crucial ways, with some type A strains having been found to be considerably more virulent in humans and laboratory animals. Cottontail rabbits have been widely implicated as a reservoir species for this subspecies; however, experimental inoculation in our laboratory revealed type A organisms to be highly virulent, resulting in 100% mortality following challenge with 50–100 organisms. Inoculation of cottontail rabbits with the same number of organisms from type B strains of bacteria was found to be rarely lethal and to result in a robust humoral immune response. The objective of this study was to characterize the protection afforded by a prior challenge with type B strains against a later inoculation with a type A strain in North American cottontail rabbits (*Sylvilagus spp*). Previous infection with a type B strain of organism was found to lengthen survival time and in some cases prevent death following inoculation with a type A2 strain of *F*. *tularensis*. In contrast, inoculation of a type A1b strain was uniformly lethal in cottontail rabbits irrespective of a prior type B inoculation. These findings provide important insight about the role cottontail rabbits may play in environmental maintenance and transmission of this organism.

## Introduction

*Francisella tularensis* (*F*. *tularensis*) is an intracellular, zoonotic bacterium, infection which induces tularemia [1]. This organism is capable of causing severe disease in a wide variety of species and, due to its low infectious dose and high virulence (the LD_50_ for some type A strains of *F*. *tularensis* has been found to be as low as one colony-forming unit (cfu) in mice), is classified as a Tier 1 Select Agent by the Centers for Disease Control and Prevention (CDC) [2, 3]. *F*. *tularensis* is classified into two subspecies: *tularensis* and *holarctica* which are referred to as type A and B respectively, and are responsible for the vast majority of human tularemia cases [4,5]. Despite a largely homologous genome, type A and type B can be readily distinguished due to large differences in virulence. Additionally, these two strains differ in global geographical distribution [6]. Type A is endemic in North America and transmission is primarily via bites from infected vectors (ticks and biting flies) or direct contact with reservoir species, such as cottontail rabbits [7–9]. Based on genetic clustering, type A strains can be further distinguished into two subpopulations: A1, primarily found in the central United States and on both coasts, and A2, which is predominantly found in the western United States [3, 7]. Furthermore, in cases of human tularemia, A1a has been associated with 4% mortality compared with 24% for A1b and 0% for A2 [10, 11]. Type B has been found in North America and is the only species endemic in Europe; this organism is associated with mosquito-borne transmission and an aquatic cycle, primarily involving beavers, muskrats, and voles [12]. These reservoir species become infected and contaminate waterways via their carcasses and urine which then serve as a route of infection for mosquito larvae, other aquatic mammals, and humans [13, 14]. Type B strains of *F*. *tularensis* cause mortality in 7% of human cases [10, 11].

Tularemia has been recognized for over a century and has long been associated with cottontail rabbits primarily due to rabbit die-offs or contact with a rabbit preceding human cases of tularemia [15, 16]. Our laboratory has previously demonstrated that type A strains are highly virulent in cottontail rabbits and challenge with 50–100 organisms results in 100% fatality within 13 days of inoculation [17]. Inoculation of type B strains rarely resulted in mortality in cottontail rabbits, and challenged rabbits elicited a robust humoral immune response through 12 weeks post-infection. Importantly rabbits challenged with type B strains appeared capable of clearing the organism [17]. The objective of this study was to determine if a prior infection with a type B strain would provide cross-protection against subsequent challenge with a type A organism in North American cottontail rabbits (*Sylvilagus spp*).

## Materials and Methods

### Ethics Statement

All aspects of this work, including experimental manipulations and sampling were conducted in strict accordance with the Guide for the Care and Use of Laboratory Animals from the National Institutes of Health and were approved by the Institutional Animal Care and Use Committee at Colorado State University (approval #13-4209A). Rabbits were trapped on public lands using Havahart traps baited with grain with approval from the Colorado Department of Natural Resources, Parks and Wildlife; cottontail rabbits are not endangered or protected. At the end of the study period, rabbits were euthanized by intravenous overdose of pentobarbital.

### Bacterial Strains and Culture Methods

All four strains of *F*. *tularensis* used in this study were provided by the CDC and were subsequently maintained in our laboratory; passage number is unknown. For simplicity, the strains are abbreviated to include the U.S. state in which they were originally isolated and their clade distinction (Table 1). MA-A1a, KY-B, and OR-B were prepared from cultures grown in Modified Mueller-Hinton (MMH) broth at 37°C with 5% CO_2_ and frozen in 15% glycerol [18]. Due to difficulty culturing the WY-A2 strain in MMH broth, cysteine heart agar with 9% chocolatized sheep blood (CHAB) was used under identical incubation settings as the strains above. Following 48 hours of growth, the agar plate was flooded with MMH broth and colonies were collected and frozen with 15% glycerol.

**Table 1:** Strains of *F*. *tularensis* used in this study.

Strain Name	Clade	Abbreviation
MA00-2987	A1b	MA-A1b
WY96-2418	A2	WY-A2
KY99-3387	B	KY-B
OR96-0246	B	OR-B

### Experimental Design and Animals

Thirty-five cottontail rabbits (16 males and 19 females) were wild-trapped along the front range of Colorado. Rabbits were transported to an ABSL-3 facility at Colorado State University and acclimatized for 2–3 weeks prior to infection. During that time they were treated for ectoparasites, a IPTT-300 temperature transponder (BioMedic Data Systems, Inc., Seaford, DE) was implanted subcutaneously under lidocaine anesthesia, and an LPS-based ELISA (see below) performed on pre-inoculation serum to provide some assurance that the rabbits were naïve. The rabbits were individually housed in standard size, stainless steel rabbit cages and provided free access to alfalfa hay, commercial rabbit pellets, and water.

Rabbits were inoculated intradermally on the right hip with 50 μL of inoculum that was confirmed by backtitration to contain between 40–80 cfu of one of two strains of *F*. *tularensis* on day 0 and one of four strains on day 28 (Table 1). On the first inoculation day (day 0), fifteen rabbits were inoculated with OR-B, fifteen rabbits were inoculated with KY-B, and five rabbits were sham inoculated with sterile phosphate buffered saline (PBS). Four weeks following this initial inoculation (day 28), six rabbits from each of the type B groups (OR-B and the KY-B) were inoculated with a type A strain, either MA-A1a or WY-A2 (Table 2). Three rabbits from each group were re-challenged with the same organism as that used for the day 0 inoculations, OR-B or KY-B. Of the five rabbits sham inoculated on day 0, two were inoculated on day 28 with MA-A1a and three were inoculated with WY-A2, to serve as positive controls. The days post-infection following the initial challenge with a type B strain are referred to as ‘dpi-1’ while days post-infection following the challenge with a type A strain are referred to as ‘dpi-2’.

**Table 2:** Challenge days, strains, and number of rabbits used.

Challenge 1 (Day 0)	Challenge 2 (Day 28)	Abbreviation	Number of rabbits
KY-B	MA-A1b	KY-B/MA-A1b	6
KY-B	WY-A2	KY-B/WY-A2	6
KY-B	KY-B	KY-B/KY-B	3
OR-B	MA-A1b	OR-B/MA-A1b	6
OR-B	WY-A2	OR-B/WY-A2	6
OR-B	OR-B	OR-B/OR-B	3
PBS	MA-A1b	PBS/MA-A1b	2
PBS	WY-A2	PBS/WY-A2	3

Body weight, temperature, and appetite of each rabbit were evaluated prior to and daily following inoculation. Weight was determined using a Pesola scale in which the rabbits were tightly wrapped in a towel and placed in a cloth bag before being suspended from the scale. Each morning the rabbits were provided a treat of peaches, pears, or pineapple and consumption was recorded. Rabbit enthusiasm for the treat proved to be an efficacious method for evaluating small changes in clinical presentation.

### Euthanasia, Necropsy, Histopathology, and Organ Burden

All rabbits were euthanized at 14 dpi-2, or earlier as necessary due to a moribund condition, which included extreme lethargy, poor appetite, hypo-responsivity, or a recumbent position. Rabbits were monitored every 12 hours for signs of progressing disease; however, despite this frequency several rabbits succumbed to death naturally due to tularemia. Gross lesions, specifically microabscessation, pulmonary consolidation, and splenomegaly were recorded for each rabbit at the time of necropsy. Due to the unavailability of control spleen weights for cottontail rabbits, splenomegaly was evaluated qualitatively based on visual appearance.

Organ burden and identity of the infecting organism were evaluated by collecting 100 mg samples of liver and spleen in a vial with 0.9mL of MMH broth containing 15% glycerol and 2 stainless steel BBs; these samples were immediately homogenized in a mixer mill and frozen at -80°C. We did not evaluate organ burdens from lung or kidney because in previous studies we found that those tissues did not provide additional information over what obtained from liver and spleen [17]. Serial ten-fold dilutions were made from 10^−1^ to 10^−3^ for the liver and spleen of rabbits challenged with the type B strains (either KY-B or OR-B) at both time points in order to quantify the organ burden. Duplicate samples (100μL) from each of the three dilutions were plated on CHAB agar plates, incubated at 37°C with 5% CO_2_ for 24–48 hours, and colony counts recorded. For each rabbit with a positive culture, DNA was extracted from a bacterial colony and PCR was used to confirm its identity as *F*. *tularensis* using a protocol described by Long et al. [19]. For rabbits sequentially inoculated with type B followed by type A strains, tissues were processed similarly, but a subspecies-specific PCR [20] was used to confirm the identity of the recovered organism.

### Serology

Rabbits were manually restrained and bled from the jugular vein prior to infection and on 14, 28, and 42 dpi-1, or upon euthanasia due to a moribund condition. In rare instances when rabbits died prior to the observation of a moribund condition (hunched/recumbent position or hypo-responsive) blood was not collected. Humoral antibody response was evaluated using an ELISA developed in our laboratory based on the World Health Organization Guidelines on Tularaemia [21] and described in detail by Brown and colleagues [17]. Briefly, 96-well plates were coated overnight at room temperature with 3 μg/mL of *F*. *tularensis* LPS obtained from BEI Resources (Manasas, Virginia, USA), rinsed and blocked with 5% non-fat dry milk. Serum samples were diluted 1:1,000 in incubation buffer and loaded in duplicate wells. Following a 1-hour incubation, the plate was emptied and rinsed. Goat anti-rabbit horseradish-peroxidase conjugate (Jackson ImmunoResearch, West Grove, Pennsylvania, USA) was used as the secondary antibody and incubated for 1-hour. The plate was again emptied and rinsed prior to the application of substrate (TMB Peroxidase Substrate, KPL, Gaithersburg, Maryland, USA) and proceeded for 15–20 minutes before the reaction was stopped via the addition of 1N hydrocholoric acid. Pooled serum from laboratory rabbits was used as a negative control.

### Statistical and Survival Analyses

Descriptive statistics and survival analysis were performed using STATA software (Stata, Statistical Software: Release 11.2, College Station, Texas). Time to death was measured in days from the time of inoculation with different strains of *F*. *tularensis*. Median survival time (in days) and 95% confidence intervals were calculated using a Kaplan Meier survival function and univariate non-parametric analyses were conducted using the log rank test to compare the survival function among cottontail rabbits infected with a combination of *Francisella* strains. Results were considered statistically significant with p-values <0.05.

## Results

Baseline body temperature was found to be between 38.3 and 39.4°C for all rabbits. Initial inoculation with either type B strain (KY-B or OR-B) resulted in elevated body temperatures starting 2–3 dpi-1 and peaking between 40.6 and 41.7°C. Overt clinical disease was not observed with the exception of one rabbit (#19) that became moribund following the initial inoculation with OR-B, and was euthanized at 8 dpi-1.

Body temperature following MA-A1b inoculation at day 0 dpi-2 appeared to have been influenced by which type B strain the rabbit had originally received. Rabbits inoculated with OR-B at the first time point were found to develop a fever 3 to 5 dpi-2, whereas rabbits that received KY-B at the first time point developed a fever 5 to 7 dpi-2. Fever associated with inoculation with WY-A2 was found to be the same for both groups and was observed 4 to 6 dpi-2. The peak body temperature for rabbits inoculated with MA-A1b and WY-A2, irrespective of which type B strain had been administered previously, was found to be between 40.6 and 41.7°C (data not shown).

The median time to euthanasia (survival time) and range following inoculation with a type A strain is summarized in Table 3. Rabbits challenged with either KY-B or OR-B at the first time point followed by MA-A1b at the second time point survived for an increased length of time (Table 3) as compared to rabbits challenged only with MA-A1b (log-rank test, p = 0.0082 and p = 0.0143, respectively). Similarly, rabbits challenged with either type B (KY-B or OR-B) strain at the first time point followed by WY-A2 at the second time point survived for an increased length of time as compared to rabbits challenged only with WY-A2 (log-rank test, p = 0.0391 and p = 0.0388, respectively).

**Table 3:** Median survival time following inoculation with a virulent type A strain after a previous inoculation with a type B strain or a sham inoculation with PBS.

Strains	Median	Survival range
KY-B/MA-A1b	7.5	7–13
KY-B/WY-A2	10	5–10
OR-B/MA-A1b *	7	5–8
OR-B/WY-A2	14	7–14
PBS/MA-A1b	4	4
PBS/WY-A2	7	5–7

Survival time for rabbits challenged only with MA-A1b was less than those inoculated first with KY/MA (p = 0.008, log rank test).

*Rabbit #19 is not included in this table; thus, the median and range for the OR/MA group is based only on 5 rabbits.

All rabbits inoculated with a type B strain followed by challenge with a type A strain at day 28 were found to have type A organism in the spleen upon necropsy (Table 4). Urine was collected from two rabbits (#4 and #26) due to the observed presence of microabscesses on the kidneys at necropsy. *F*. *tularensis* was cultured in the urine from each rabbit, type A and type B, respectively. Rabbits challenged with type B at both time points were found to have cleared the organism from the liver and spleen upon euthanasia at the end of the study period, with an exception for one rabbit (#13) that was found to have 700 organisms/gram in the spleen.

**Table 4:** Summary of clinical response, pathology, and organism isolated following inoculation with a type A strain.

Strains	Rabbit	DPI-2 Euthanized	Splenomegaly?	Microabscess-ation?	Organism Type Isolated
**KY-B/MA-A1b**	1	8	-	+	A
	2	7	-	+	A
	3	7	-	+	A
	4	13	+	+	A
	5	8	-	+	A
	6	7	-	+	A
**KY-B/WY-A2**	7	10	-	-	A
	8	14	-	-	A
	9	10	-	-	A
	10	5	+	-	A
	11	9	-	-	A
	12	10	+	+	A
**KY-B/KY-B**	13	14	-	-	B
	14	14	-	-	-
	15	14	-	-	-
**OR-B/OR-B**	16	14	-	-	-
	17	14	+	-	-
	18	14	-	-	-
**OR-B/MA-A1b**	19	8*	+	-	NA
	20	7	+	+	A
	21	5	+	+	A
	22	8	-	+	A
	23	6	+	+	A
	24	7	-	+	A
**OR-B/WY-A2**	25	14	-	-	A
	26	7	+	+	A
	27	7	+	+	A
	28	14	-	-	A
	29	14	-	+	A
	30	14	+	-	A
**PBS/WY-A2**	31	5	+	-	A
	32	7	+	-	A
	33	7	-	-	A
**PBS/MA-A1b**	34	4	-	+	A
	35	4	-	+	A

*The dpi euthanized for this rabbit are associated with days following the first inoculation (dpi-1), as this rabbit succumbed to infection following the initial inoculation with the type B strain (OR-B). NA = not applicable.

The primary gross lesions observed were microabscesses on the liver or spleen and splenomegaly (Table 4). Rabbits inoculated with MA-A1b at either time point were found to have a much higher incidence of microabscessation upon necropsy than rabbits challenged with WY-A2 (OR = 35.8, 95% CI = 3.47–368.8, p = 0.0027). Splenomegaly occurred at the same frequency in rabbits, irrespective of inoculation with MA-A1b or WY-A2 (OR = 0.635, 95% CI = 0.143–2.82, p = 0.5505). Consistent with previous work completed in our laboratory, lung consolidation was observed in two of the rabbits inoculated with OR-B at both time points (data not shown).

**Fig 1:**
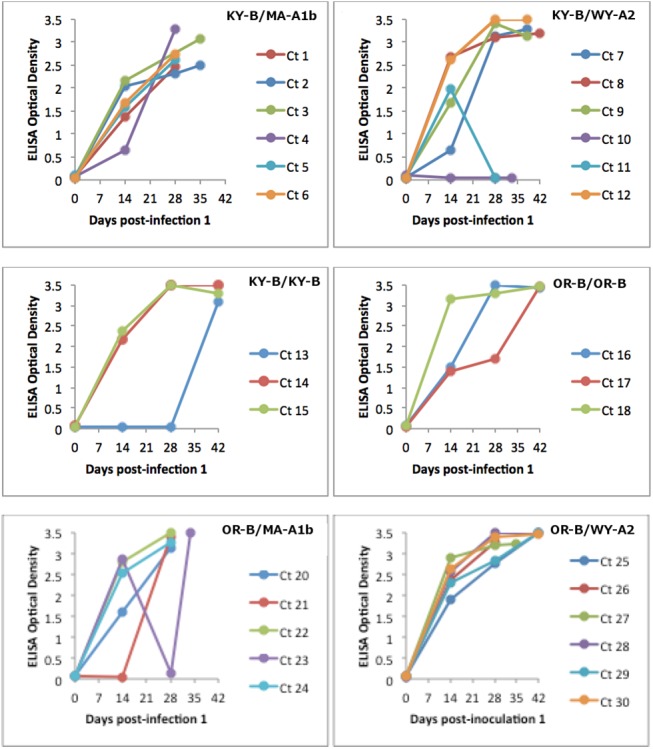
ELISA antibody responses of rabbits infected with a combination of *F*. *tularensis* strains. Rabbits were inoculated with *F*. *tularensis* on days 0 and 28.

With a few exceptions, rabbits developed a robust antibody response characterized by a moderate rise in antibodies by 14 dpi-1 which peaked at 28 dpi-1 and remained stable until euthanasia at 42 dpi-1 or earlier (Fig 1). Serologic responses from several rabbits appeared aberrant; for example, antibodies were not detected for rabbit #10 at any time point following inoculation and rabbit #11 was found to have an antibody response at 14 dpi-1 but not at at 28 dpi-1. We re-assayed all of these samples, obtained the same result, and were unable to explain these apparent discrepancies. Rabbit #13 was not found to have antibodies until 42 dpi-1; however, the magnitude of the response at that time point was similar to other rabbits of the same group. Rabbit #21 did not have detectable antibodies at 14 dpi-1 but was found to have response equivalent to others in its group by 28 dpi-1. Rabbit #23 seroconverted by 14 dpi-1 but antibodies were not detected at 28 dpi-1; however, upon euthanasia at 34 dpi-1 the antibody response was found to be comparable to the other rabbits in the group.

## Discussion

This study provided an initial characterization in cottontail rabbits of the effect of a prior type B infection on the outcome of subsequent inoculation with a virulent type A strain of *F*. *tularensis*. Infection of laboratory mice with both type A and B strains results in uniform mortality [4] and it was of interest to evaluate sequential infections in a natural host species. Understanding how a prior inoculation with a low virulent strain of this organism (type B), resulting in a robust humoral immune response, impacts host survival following exposure to a highly virulent strain of *F*. *tularensis* has important implications for further understanding the role of cottontail rabbits in the maintenance and transmission of *F*. *tularensis*. Clearly, humoral immunity *per se* likely does not mediate protective immunity to an intracellular pathogen like *F*. *tularensis*, but was used in this study as an index of immune response to infection.

Our study establishes that a robust immune response, as assessed by antibody production, initiated by challenge with a type B strain of *F*. *tularensis* is, in some cases, sufficient to lengthen the survival time following infection with a virulent type A strain. Inoculation with MA-A1b was uniformly lethal prior to 14 dpi regardless of whether or not the rabbit was previously inoculated with a type B strain; however, rabbits that were previously exposed were found to survive for a longer duration as compared to those that received PBS at the first time point. Inoculation with WY-A2 alone was found previously [17] to cause uniform mortality in cottontail rabbits; however, when WY-A2 was delivered subsequent to a challenge with either KY-B or OR-B, we observed lengthened survival periods in some rabbits and complete protection from mortality in others (n = 4) during the first 14 days following inoculation. This finding is not altogether surprising as A1b and A2 have very different mortality ratios in humans, 24% and 0%, respectively; which is suggestive of differences in ability to colonize the host, capability to evade the immune system, or both [3].

Interestingly, the two rabbits (#26 and #27) that were euthanized from the OR/WY group prior to 14 dpi-2) were found to have antibody levels similar to the rabbits that survived which, as has been previously observed, provides an additional indication that antibody production is not the major influence on host outcome following exposure to *F*. *tularensis*. This observed difference indicates that a robust antibody response is associated with partially protecting rabbits from inoculation with a virulent strain of *F*. *tularensis* (WY-A2), although is insufficient to afford protection against highly virulent strains (MA-A1b).

The pattern and magnitude of the antibody response proved to be fairly uniform irrespective of the inoculating strain and length of survival following inoculation. Sequential exposure to a type B strain at days 0 and 28 was found to result in a humoral immune response of a similar magnitude to those that received only one inoculation with a type B strain. Additionally, fever was observed between 3 and 7 dpi in the majority of rabbits regardless of the inoculated strain. This febrile response was similar to that described following aerosol exposure of New Zealand White rabbits with the Schu S4 strain of *F*. *tularensis*. [22].

Surprisingly, all rabbits inoculated with type A strains were found to have spleens that were culture positive for this organism (either MA-A1b or WY-A2) at the time of necropsy, even in instances where the rabbit survived until 14 dpi-2. Because both type A strains have been found to be 100% fatal when administered solely, the finding of culture positive tissues in apparently healthy animals is suggestive of an important role for the adaptive immune response.

This study was subject to some limitations. First, the cottontail rabbits utilized in this study were wild-caught and thus, were undoubtedly harboring various organisms that could influence the immune responses observed in our study. However, our intent was to study *F*. *tularensis* in its natural host and thus, we believe our findings to be representative of a natural setting. Secondly, the likelihood in a natural setting of a sequential infection in cottontail rabbits of a type B strain of *F*. *tularensis* followed by a type A strain is unknown. These strains do overlap geographically and thus, it is certainly a possibility that a single animal could be infected with several strains of the organism. Next, captivity for cottontail rabbits is indisputably a stressful environment. We attempted to alleviate the stress of the laboratory setting by handling the rabbits gently and infrequently. Finally, chocolate agar plates were used only for the WY-A2 strain due to growth requirements which may have confounded the culture results.

Our results strongly suggest that although a previous exposure to a type B strain of *F*. *tularensis* does not provide full protection against challenge with a virulent type A strain, it does lengthen the survival period for rabbits inoculated with either KY-B or OR-B followed by WY-A2 and in some cases, rabbits survive infection altogether. These findings are important and help to shape our understanding of the role that cottontail rabbits may play in the maintenance and transmission of *F*. *tularensis* amongst humans and other animals.
